# Evaluation of late cardiac effects after multisystem inflammatory syndrome in children

**DOI:** 10.3389/fped.2023.1253608

**Published:** 2023-08-24

**Authors:** Rik De Wolf, Mahmoud Zaqout, Kaoru Tanaka, Laura Muiño-Mosquera, Gerlant van Berlaer, Kristof Vandekerckhove, Wendy Dewals, Daniël De Wolf

**Affiliations:** ^1^ Department of Pediatric Cardiology, University Hospital Brussels, Brussels, Belgium; ^2^ Department of Pediatric Cardiology, Antwerp University Hospital, Antwerp, Belgium; ^3^ Department of Pediatric Cardiology, ZNA Queen Paola Children’s Hospital, Antwerp, Belgium; ^4^ Department of Radiology, University Hospital Brussels, Brussels, Belgium; ^5^ Department of Pediatric Cardiology, Ghent University Hospital, Ghent, Belgium; ^6^ Department of Pediatric Intensive Care, University Hospital Brussels, Brussels, Belgium

**Keywords:** MIS-C, CMR, pediatric, SARS-CoV-2, children, outcome, cardiac

## Abstract

**Introduction:**

Multisystem inflammatory syndrome in children (MIS-C) is associated with important cardiovascular morbidity during the acute phase. Follow-up shows a swift recovery of cardiac abnormalities in most patients. However, a small portion of patients has persistent cardiac sequelae at mid-term. The goal of our study was to assess late cardiac outcomes of MIS-C.

**Methods:**

A prospective observational multicenter study was performed in children admitted with MIS-C and cardiac involvement between April 2020 and March 2022. A follow-up by NT-proBNP measurement, echocardiography, 24-h Holter monitoring, and cardiac MRI (CMR) was performed at least 6 months after MIS-C diagnosis.

**Results:**

We included 36 children with a median age of 10 (8.0–11.0) years, and among them, 21 (58%) were girls. At diagnosis, all patients had an elevated NT-proBNP, and 39% had a decreased left ventricular ejection fraction (LVEF) (<55%). ECG abnormalities were present in 13 (36%) patients, but none presented with arrhythmia. Almost two-thirds of patients (58%) had echocardiographic abnormalities such as coronary artery dilation (20%), pericardial effusion (17%), and mitral valve insufficiency (14%). A decreased echocardiographic systolic left ventricular (LV) function was detected in 14 (39%) patients. A follow-up visit was done at a mean time of 12.1 (±5.8) months (range 6–28 months). The ECG normalized in all except one, and no arrhythmias were detected on 24-h Holter monitoring. None had persistent coronary artery dilation or pericardial effusion. The NT-proBNP level and echocardiographic systolic LV function normalized in all patients, except for one, who had a severely reduced EF. The LV global longitudinal strain (GLS), as a marker of subclinical myocardial dysfunction, decreased (*z* < −2) in 35%. CMR identified one patient with severely reduced EF and extensive myocardial fibrosis requiring heart transplantation. None of the other patients had signs of myocardial scarring on CMR.

**Conclusion:**

Late cardiac outcomes after MIS-C, if treated according to the current guidelines, are excellent. CMR does not show any myocardial scarring in children with normal systolic LV function. However, a subgroup had a decreased GLS at follow-up, possibly as a reflection of persistent subclinical myocardial dysfunction.

## Introduction

Children affected with a SARS-CoV-2 infection usually have a mild clinical disease course (1). However, in April 2020, a hyperinflammatory multisystem syndrome was described, which occurred 2–6 weeks after SARS-CoV-2 exposure. It was referred to as multisystem inflammatory syndrome in children (MIS-C). Up to 80% of MIS-C patients have cardiovascular involvement, including signs of myocarditis with an elevated N-terminal prohormone of brain natriuretic peptide (NT-proBNP) and a decreased systolic left ventricular (LV) function, coronary artery dilation, pericardial effusion, arrhythmias, and conduction abnormalities (2–6). Although the follow-up demonstrated a swift recovery of cardiac abnormalities and left ventricular function in most patients, a small number had persistent cardiac sequelae at mid-term, including a decreased left ventricular ejection fraction (LVEF) (7–9), subclinical myocardial damage illustrated by reduced LV strain (10), and myocardial fibrosis on CMR (11). The goal of our study was to assess late cardiac outcomes after hospitalization for MIS-C using NT-proBNP, echocardiography, 24-h Holter monitoring, and CMR.

## Materials and methods

We performed a prospective observational multicenter study in patients aged <18 years diagnosed with MIS-C according to WHO criteria (12) between May 2020 and August 2022 in one of the following Belgian centers: Brussels University Hospital, ZNA Queen Paola Children's Hospital, Ghent University Hospital, and Antwerp University Hospital. The exclusion criteria were the presence of pre-existing myocardial dysfunction and the need for general anesthesia to perform cardiac MRI, as the risk would outweigh the benefit. The follow-up visit was organized at least 6 months after hospitalization for MIS-C. From a total of 62 patients who met the inclusion criteria, 36 were included, 18 declined to participate, and 8 could not give informed consent because of a language barrier. Written informed consent was given by the parents of the participating patients. The study protocol was approved by the Ethics Committees of all participating centers (EC 2021-115/B.U.N. 1432021000463).

### Patient data

All demographical, clinical, biochemical, and echocardiographic data were collected in a standardized form. Demographic (sex, ethnicity, and age at admission), history (recent infection with SARS-CoV-2 or close contact in the weeks before presentation), anthropometric [height, weight, and body mass index (BMI)], and clinical (need for ICU admission, inotropic support, and treatment regime) data were collected at baseline (admission for MIS-C).

### Laboratory investigations

All patients underwent SARS-CoV-2 testing at admission by polymerase chain reaction on nasopharyngeal swabs. Previous SARS-CoV-2 exposure was detected through IgG serological assessment. Biochemical data included cardiovascular disease biomarkers (NT-proBNP and troponin) and D-dimers. NT-proBNP measurement was repeated at the follow-up visit, simultaneously with the peripheral intravenous cannulation necessary to perform the CMR with gadolinium administration.

### Echocardiographic and electrocardiogram data

Echocardiography was performed by a single pediatric cardiologist in each participating center at baseline and at follow-up. The echocardiographic parameters recorded included the presence of pulmonary hypertension (PH), pericardial effusion, coronary dilation, and valvular insufficiency as well as cardiac dimensions and function. Coronary dilation was defined as a *z*-score of ≥2.0 (13). Cardiac function was evaluated by left ventricular fractional shortening (FS) (M-mode) and left ventricular ejection fraction (Simpson biplane method). A decreased cardiac function was defined as an FS of < 28% or EF of < 55%. Left ventricular end-diastolic diameter (LVEDD) and left ventricular end-systolic diameter (LVEDs) and their respective *z*-scores were recorded. An evaluation of LV regional wall abnormalities and subclinical LV dysfunction by global longitudinal strain (GLS) measurement through speckle tracking echocardiography (STE) was performed at follow-up. It was added to the study at a later stage; thus, results are only partially available for the cohort. Three apical views (four-chamber, two-chamber, and three-chamber) were used. GLS calculation was performed offline using a semiautomatic method (GE EchoPac Software, GE Healthcare, USA). The borders of the studied LV myocardial segments were adjusted manually. The peak longitudinal strain values for each view were averaged to calculate LV GLS. An adjustment for body surface area was performed and represented by the *z*-score as described by Dallaire et al. (14). A decreased GLS was defined as a *z*-score of <−2.

A standard 12-lead electrocardiogram (ECG) was performed during admission for MIS-C and was repeated, combined with a 24-h Holter monitoring at follow-up, to detect arrhythmia as a possible late complication of MIS-C.

### CMR-imaging

A standard CMR protocol was performed using a Philips Achieva dStream 1.5T or Ingenia 3.0T scanner (Philips Medical Systems, Best, Netherlands). SSFP-cine sequences were performed for LV and RV measurements. Tissue characterization before contrast was performed with T1 and T2 mapping to visualize myocardial injury and edema, respectively. A T2-weighted short tau inversion recovery sequence was used to visualize edema. Late gadolinium enhancement (LGE) imaging was performed 10 min after an intravenous administration of 0.1 mmol/kg gadoterate meglumine (Dotarem, Guerbet, Villepinte, France). Cardiac volumes and mass of the left and right ventricles were analyzed using Circle Cardiovascular Imaging Inc. (Calgary, Canada). An increased left ventricular end-diastolic volume adjusted for body surface area (ml/m^2^) was defined as an LVEDD of >p97 using pediatric CMR reference values (15).

### Statistical analysis

Statistical analysis was done with IBM SPSS Statistics 28.0 (IBM Corp., Armonk, NY, USA). Shapiro–Wilk test and histogram were used to test the normality for each variable. Continuous variables are expressed as mean ± standard deviation or median and interquartile range (25th–75th percentile) as appropriate. Discrete variables are expressed as numbers and percentages. A comparison of characteristics between the different patient groups was done by using the Mann–Whitney *U* test for continuous variables and Fisher's exact test for dichotomous variables.

## Results

### Patients

We included 36 children with MIS-C. Patient characteristics at baseline are outlined in Table 1. The median age at admission was 10.0 (8.0–11.0) years, and among them, 21 (58%) were girls. Most patients had Caucasian descent (69%). A body mass index (BMI) of >25 was observed in three (8%) patients. Comorbidity was limited to one patient with familial Mediterranean fever and one with pre-existing renal failure. All except one received immunoglobulins and/or corticosteroids as MIS-C treatment. Treatment with low-dose aspirin was started in all patients. ICU admission was warranted in 29 patients (81%), with almost half of them (48%) needing inotropic support for a mean duration of 3.6 ± 0.6 days.

**Table 1:** Clinical patient characteristics.

	*n* = 36
Age at admission (years)	10.0 (8.0–11.0)
Sex (Female)	21 (58)
Height (cm)	141.5 ± 2.4
Weight (kg)	34.5 (25.0–49.3)
BMI (kg/m^2^)	17.8 (14.6–19.9)
Ethnicity	
Caucasian	25 (69)
African	9 (25)
Asian	2 (6)
COVID status	
IgG+	25 (69)
PCR+	4 (11)
Contact	4 (11)
IgG and PCR+	3 (8)
Treatment regime	
Immunoglobulins	13 (36)
Immunoglobulins + corticosteroids	21 (58)
Corticosteroids	1 (3)
None	1 (3)
ICU admission	29 (81)
Inotropic support	14 (39)
Duration of inotropic support (days)	3.6 ± 0.6

Values are presented as *n* (%), means ± SD, or median (Q1–Q3). BMI, body mass index; ICU, intensive care unit; IgG, immunoglobulin G; PCR, polymerase chain reaction.

### Cardiac assessment at MIS-C admission

The cardiac assessment was performed routinely during MIS-C admission, and the results are presented in Table 2. Cardiac biomarkers measurement showed significantly elevated NT-proBNP and D-dimers in all patients and an elevated troponin level in 28 (78%).

**Table 2:** Cardiac findings at baseline.

	*n* = 36
Biomarkers	
D-dimer (ng/ml)	3,368.5 (1,565.0–4,781.8)
Troponin (ng/L)	48.5 (15.5–146.0)
NT-proBNP (pg/ml)	5,125.0 (1,505.0–20,153.0)
Echocardiographic findings	
Decreased LV function	21 (58)
LVEF (%)	57.0 ± 1.9
FS <28%	13 (37)
FS (%)	29.0 ± 1.2
LVEF <55%	14 (39)
LVEDD *z*-score >2 (%)	1 (3)
Echocardiographic abnormality at diagnosis	21 (58)
Coronary artery dilation	20 (56)
Pericardial effusion	6 (17)
Mitral valve insufficiency	5 (13)
Pulmonary hypertension	3 (8)
Left ventricular hypertrophy	
ECG abnormalities	13 (36)
Repolarization abnormalities	7 (19)
QTc prolongation	3 (8)
Left ventricular hypertrophy	2 (6)
First-degree AV block	1 (3)
Sinus bradycardia	1 (3)

Values are presented as *n* (%), means ± SD, or median (Q1–Q3). NT-proBNP, N-terminal prohormone of brain natriuretic peptide; LVEF, left ventricular ejection fraction; FS, fractional shortening; LVEDD, left ventricular end-diastolic diameter; AV block, atrioventricular block.

A total of 13 patients (36%) had ECG abnormalities during admission. More than half of those had repolarization abnormalities (ST segment elevation, flattened or inverted T waves), three (8%) had QTc prolongation, two (6%) had left ventricular hypertrophy, one (3%) had sinus bradycardia despite inotropes, and one (3%) had a first-degree AV block. None demonstrated supraventricular or ventricular arrhythmias.

All patients underwent an echocardiographic evaluation, and abnormalities were present in almost two-thirds of patients (58%). A total of 20 patients (56%) had dilated coronary arteries, but none had more than small coronary artery aneurysms (*z*-score <5). Pericardial effusion was observed in a small portion of patients (17%), none of which was hemodynamically relevant. A total of five patients (14%) had mitral valve insufficiency (MI), three (8%) had pulmonary hypertension, and three (8%) had septal hypertrophy.

An evaluation of left ventricular function was performed by calculating FS, LVEF, and LVEDD. A total of 13 patients (36%) had both a diminished LVEF and FS in combination with an LVEDD *z*-score of >2 in 1 (3%) patient. One had a decreased LVEF and a borderline FS of 29%. The LV dysfunction was mostly mild (71%), but some had moderate (21%) or severe (8%) impaired systolic LV function. An LVEDD *z*-score of >2 was present in one patient (3%) only.

### Cardiac assessment at the follow-up visit

The mean time interval to the follow-up visit was 12.1 ± 5.8 months (range 6–28 months). The follow-up data are outlined in Table 3. The ECG was normal in all except one patient with persistent repolarization abnormalities. A 24-h Holter monitoring showed no pathological supraventricular or ventricular arrhythmias. An echocardiographic evaluation of persistent cardiac abnormalities or decreased LV function was performed in all patients. None had persistent coronary artery dilation or pericardial effusion. Mitral valve insufficiency and PH normalized in patients with a normal systolic LV function. One patient showed persistent septal hypertrophy. The echocardiographic LV systolic function was still impaired in only one patient with an extremely elevated NT-proBNP. In this patient, echocardiographic parameters showed an increased LVEDD (*z*-score +5), a diminished LVEF of 10%, and an FS of 8% with consequent mitral valve insufficiency and PH.

**Table 3:** Cardiac findings at follow-up.

	*N* = 36
Follow-up time (months)	12.1 ± 5.8
NT-proBNP (pg/ml)	35.0 (24.8–51.0)
Echocardiographic findings	
Echocardiographic evaluation of LV function	
LVEF (%)	64.9 ± 1.8
LVEF <55%	1 (3)
FS (%)	35.6 ± 1.1
FS <28%	1 (3)
LVEDD *z*-score >2	1 (3)
GLS (%, *n* = 14)	−18.2 ± 0.7
Echocardiographic abnormality at follow-up	2 (6)
Coronary artery dilation	0
Pericardial effusion	0
Mitral valve insufficiency	1
Pulmonary hypertension	1
Left ventricular hypertrophy	1
Abnormal Holter findings	0
ECG abnormalities	1 (3)
Repolarization abnormalities	1 (3)
CMR findings	
LVEF	60.7 ± 10.2
LVEF <55%	3 (9)
LVEDD (ml/m^2^)	78.3 ± 24.1
LVEDD >p97	3 (9)
Fibrosis	1 (3)

Values are presented as *n* (%), means ± SD, or median (Q1–Q3). NT-proBNP, N-terminal prohormone of brain natriuretic peptide; LVEF, left ventricular ejection fraction; FS, fractional shortening; ECG, electrocardiogram; LVEDD, left ventricular end-diastolic diameter.

A measurement of the global LV strain was added to detect subclinical LV dysfunction later during follow-up. The GLS was calculated in 14 patients, and 5 (35%) had a decreased GLS percentage adjusted for BSA (*z*-score <−2; range −3.4/−2.5). The distribution of the affected LV myocardial regions in patients with a decreased GLS was variable and different. All children with a decreased GLS were admitted to the ICU during the acute phase, and two had a decreased echocardiographic LV function. However, only one needed inotropic support. Three patients had an increased troponin level, and coronary arteries were dilated in four children. None had an abnormal baseline ECG. We found no correlation between children with either normal or decreased GLS regarding the need for inotropic support, PICU admission, increased cardiac biomarkers, decreased cardiac function, or any other cardiac abnormalities at baseline. All of these patients had normal biochemical, echocardiographic (except for a decreased GLS), and Holter results at follow-up.

A CMR was performed in 35 patients at follow-up. In one patient with claustrophobia, the CMR could not be obtained. The CMR LVEF decreased (LVEF <55%) in three patients, of which one had a severely reduced LVEF of 10%. This was the only patient with LGE as a sign of myocardial fibrosis. This 16-year-old patient was admitted to our hospital with heart failure when the pandemic was first reported (April 2020), 6 weeks after a SARS-CoV2 infection needing respiratory support. He was our first MIS-C patient and did not receive immunoglobulins or corticosteroids. Heart transplantation was necessary due to the severity of the heart failure, despite maximal medical treatment. The patient is now doing well. The two other patients had a mild reduced LVEF on CMR (45%–55%) and no signs of fibrosis, and the echocardiographic evaluation of systolic LV function was normal. LVEDD was >p97 in three children of which one is the patient described above. The CMR LVEF and echocardiographic LV function and dimensions were normal in the remaining two. All patients with a decreased echocardiographic GLS had a normal CMR.

## Discussion

In this Belgian multicenter prospective observational study, we report on the late cardiac outcomes of 36 children who were recruited during the acute phase of an MIS-C episode. In the late-term follow-up visit, with the evaluation of late cardiac outcomes through NT-proBNP measurement, ECG and 24-h Holter monitoring, echocardiography, and CMR performed after a mean period of 1 year with a maximal interval of 28 months after MIS-C admission. To the best of our knowledge, this includes the longest follow-up interval of a multimodal cardiac evaluation, including CMR, after MIS-C.

### Echocardiographic abnormalities

As in Kawasaki disease, coronary dilatation is a common finding in the acute phase of MIS-C with a prevalence rate ranging from 7% to 35%. Most patients have coronary dilatation or small coronary artery aneurysms. Giant coronary aneurysms are rare. The follow-up demonstrates a regression of the coronary artery dilatation to 79% after 30 days and up to 100% after 90 days, although there are reports of persistent giant coronary artery aneurysms (4, 6, 11, 16, 17). In our cohort, a relatively large number (56%) had coronary artery dilation, but none had more than small coronary aneurysms, defined as a *z*-score of <5. It is suggested that fever and tachycardia caused by the hyperinflammatory state are responsible for the frequent mild coronary artery dilation and not the inflammatory damage to the arterial wall itself, as seen in Kawasaki disease (18). This could also explain the rapid normalization of the coronary artery dimension in our cohort. As there is a significant overlap between the symptomatology of MIS-C and Kawasaki disease, it is possible that some of the children with persistent important coronary aneurysms reported in the literature had incomplete Kawasaki disease rather than MIS-C.

Pericardial effusion occurs in up to 28% of patients in the acute phase of MIS-C and resolves quickly (19). Severe effusion with tamponade necessitating pericardiocentesis is very rare (11). In our cohort, 17% had a pericardial effusion, but none needed percutaneous drainage nor showed pericardial effusion at follow-up echocardiography or CMR.

Mitral valve incompetence is associated with MIS-C, possibly secondary to a decreased LV function with LV dilation or as a sign of valvulitis. Valverde et al. (6) describe a prevalence of MI in 42% of patients at diagnosis. In our population, MI was present in five patients (13%). Four had a decreased LV function and one had a concomitant increased LVEDD. An isolated MI, representing valvulitis, was observed only in one patient and did not persist at follow-up. MI was present at follow-up in the patient with persistent LV dysfunction and dilation.

PH was seen in three patients of whom one had a decreased systolic LV function, one had mitral valve insufficiency, and one had isolated PH. None had interference of any pulmonary involvement (pneumonia, atelectasis, and pleural effusion). Isolated PH is not known to be an expected condition in MIS-C. In our patients, it can be hypothesized that both pulmonary vascular reactivity, caused by systemic inflammation, and LV dysfunction could be the cause for this increased pulmonary arterial pressure. Only the patient with a severely impaired LV systolic function had a postcapillary PH at follow-up.

### ECG abnormalities

ECG abnormalities are associated with MIS-C and are observed in up to two-thirds of patients (3, 11). This study showed ECG abnormalities in 36% of children, mainly during repolarization. Myopericarditis with consequent myocardial inflammation and edema seems to be responsible for these conduction abnormalities, a hypothesis supported by the normalization of the ECG in most cases after MIS-C recovery (20). Tachyarrhythmias are rare in MIS-C. Dionne et al. (2) reported a 1.7% prevalence in 2,343 patients <21 years of supraventricular tachycardia, accelerated junctional rhythm, or ventricular tachycardia. In our patient group, we did not observe any tachyarrhythmias during admission nor at the 24-h Holter monitoring during follow-up.

### Echocardiographic evaluation of cardiac function

An impaired echocardiographic systolic LV function is observed in 31%–63% of MIS-C patients at diagnosis. An ICU admission and the need for inotropic support are not uncommon, and a small number even need extracorporeal membrane oxygenation (21, 22). However, recovery of LV systolic function on echocardiography is observed in most patients already at short-term follow-up. Persistence of LV dysfunction at mid-term follow-up is rare (7). In our cohort, 39% of patients had a decreased LVEF during MIS-C, all needing inotropic support. None required ECMO. Our follow-up data confirm the lasting recovery of systolic LV function at late-term. Severely impaired systolic LV function was observed in one patient at follow-up.

As LVEF is a marker of clinical myocardial function, LV GLS represents both clinical and subclinical myocardial performance. In MIS-C, LV GLS is significantly reduced, and mainly the basal LV segments seem to be affected. The decreased GLS seems to persist longer than a diminished LVEF. The latter is usually recovered at the 2-month follow-up, while GLS improves especially between 2 and 6 months after disease onset (10, 23, 24). It is suggested that a diminished GLS could reflect persistent subclinical myocardial damage. In our study, 5 out of 14 patients (35%) had a GLS *z*-score of <−2 at follow-up. The FS, LVEF, and CMR were normal in all patients. We could not identify any differences in characteristics at baseline in these groups. Thus, it remains uncertain which patients are at greater risk for a decreased GLS during long-term follow-up, as even children with normal LVEF are at risk (25). Although GLS has proved its value in the detection of subclinical myocardial dysfunction, further investigation is necessary to assess the clinical significance and predictive value of a decreased LV GLS during MIS-C follow-up. We believe that follow-up needs to be continued in these patients with diminished GLS until normalization.

### CMR

CMR data in the acute phase of MIS-C are scarce. Myocardial edema and LGE as signs of myocardial fibrosis are present in 14%–35% (6, 26). In a multicenter study by Aeschlimann et al. (27), 18% of patients met the Lake Louise criteria for myocarditis at a median time of 28 days after the onset of symptoms. It seems clear that myocarditis can be present during the hyperinflammatory state in MIS-C and can persist for a short term in some patients. Emerging mid-term follow-up CMR data after MIS-C have become available, but conflicting results are being published. Bartoszek et al. (28) could not identify any CMR abnormalities at the 3-month follow-up in 19 children with cardiac involvement during the acute phase of MIS-C, and neither could Barris et al. (3) at the 9-month follow-up. These results contrast with other data that show the presence of LGE, without LV dysfunction or any other clinically significant cardiac abnormalities, in up to 50% after a mean follow-up of 9 months (11, 29). We performed a CMR after a mean period of 12 months with a range of 6–28 months and observed normalization of the cardiac function in almost all patients. The patient described above, who did not receive adequate MIS-C treatment because of a late recognition in the early stages of the pandemic, had a severely reduced EF (10%) and an increased LVEDD (197 ml/m^2^) and LGE on CMR 6 months after MIS-C. Interestingly, two other patients had mildly reduced EF on CMR and normal echocardiographic LVEF and FS. The discrepancy between the evaluation of cardiac function on echocardiography and CMR is not clearly understood. Possibly, CMR, as the gold standard for LV function evaluation, is more sensitive for detecting a subtle decreased LV function. CMR LVEDD was >p97 in two patients with normal echocardiographic LVEDD and LV function.

It can be hypothesized that CMR at mid-term follow-up still shows persistent anomalies that may recover over time. Sirico et al. (10) described 6 patients with LGE on initial CMR 18 days after the disease onset, who underwent repeat CMR 6 months later. Although LGE was still present in 5 out of 6 patients, reduced extension was observed. Benvenuto et al. (30) describe a similar patient with LGE on CMR 2 months after MIS-C, with an improvement on repeat CMR at the 9-month follow-up. CMR can be a useful tool during MIS-C follow-up; however, the timing is still open for discussion. Timing CMR 3–6 months after MIS-C diagnosis can be performed to assess whether participating in competitive sports is safe, in addition to an exercise stress test and/or 24-h Holter monitoring. However, if echocardiography is normal, it could be considered to evaluate for myocardial fibrosis at a later stage during follow-up, as the degree of LGE seems to improve over time. CMR even seems unnecessary in most children, as no relevant cardiac abnormalities were found in patients with LGE on CMR and with normal echocardiographic cardiac function. Our findings seem to support this idea as no LGE was observed after a long mean follow-up period of 12 months in patients who received adequate MIS-C treatment.

### Limitations

There are some limitations in our study. First, although the mean follow-up duration is one of the longest reported, it occurred not at a fixed time interval after MIS-C admission because of various factors such as the availability of CMR during the pandemic. Second, our LV GLS results could be biased as we added evaluation for subclinical myocardial damage through LV GLS at a later stage of the study course. Finally, we could include only 36 out of 62 MIS-C patients; however, this has not significantly changed our results because the standard follow-up in those children who did not participate in the study did not show cardiac abnormalities.

## Conclusion

Late cardiac outcomes after MIS-C, if treated according to current guidelines, are excellent. CMR does not show any myocardial scarring in children with a normal echocardiographic LVEF. Subclinical myocardial damage can persist in the late term, and further follow-up seems appropriate in these patients. However, the clinical significance of these findings has yet to be determined.

## Data Availability

The raw data supporting the conclusions of this article will be made available by the authors without undue reservation.
